# Multizonal intraepithelial neoplasia of the lower genital tract and anus in women: terminology for defining the disease, an introduction by the British Society for Colposcopy and Cervical Pathology (BSCCP), International Anal Neoplasia Society (IANS), European Federation for Colposcopy (EFC) and British Society for the Study of Vulval Disease (BSSVD) scientific committees

**DOI:** 10.1038/s41416-025-03259-z

**Published:** 2025-12-09

**Authors:** Sarah J. Bowden, Laura Burney Ellis, Tamzin Cuming, Danielle Brogden, Susan M. Sherman, David A. Finch, Shaun Haran, Julie Bowring, George Mochloulis, Alice M. Schofield, Archie Krishna, Andrew G. Renehan, Maggie E. Cruickshank, Pierre Martin-Hirsch, Maria Kyrgiou, Deirdre Lyons, Theresa Freeman-Wang

**Affiliations:** 1 Institute of Reproductive and Developmental Biology, Department Digestion, Metabolism, & Reproduction – Surgery & Cancer, Imperial College London, London, UK; 2 Imperial College NHS Trust, London, UK; 3 Homerton Anogenital Neoplasia Service, Homerton University Hospital London, London, UK; 4 Surgery & Cancer, Imperial College London, London, UK; 5 Department of Colorectal Surgery. Maidstone and Tunbridge Wells NHS Trust, Tunbridge Wells, UK; 6 School of Psychology, University of Sheffield, Sheffield, UK; 7 Manchester Cancer Research Centre (MCRC), NIHR Manchester Biomedical Research Centre, Division of Cancer Sciences, School of Medical Sciences, Faculty of Biology, Medicine and Health, University of Manchester, Manchester, UK; 8 Mount Vernon Cancer Centre, East & North Herts NHS Trust, Rickmansworth, UK; 9 Department of Gynaecological Oncology, The Christie NHS Foundation Trust, Manchester, UK; 10 University Hospitals Birmingham, Birmingham, UK; 11 Department of Colorectal Surgery, The Christie NHS Foundation Trust, Manchester, UK; 12 Centre for Women’s Health Research, Aberdeen Maternity Hospital, University of Aberdeen, ABERDEEN, UK; 13 NIHR Clinical Research Facility, Lancashire Teaching Hospitals NHS Trust, Lancashire, UK; 14 Department of Obstetrics & Gynaecology, Whittington Hospital, London, UK

**Keywords:** Education, Cancer prevention, Risk factors

## Abstract

Multizonal anogenital intraepithelial neoplasia (MZIN) is an uncommon chronic pre-malignant condition. In the United Kingdom (UK) and elsewhere MZIN is managed by a variety of clinical specialities with differing strategies, resulting in a lack of standardisation in diagnosis and treatment which ultimately disadvantages those affected. Screening for anogenital precancerous conditions is sporadic rather than nationalised in the UK and elsewhere in Europe, with the exception of the cervix. To address this lack of standardisation, the BSCCP brought together a panel of stakeholders from aligned expert society committees (IANS, EFC and BSSVD) to review existing evidence and provide a framework for national UK guidelines. Here, we define terminology and scope, as a platform for subsequent guideline development and guide further research. We define MZIN as Human Papillomavirus (HPV)-related squamous intraepithelial lesions occurring in two or more anogenital regions. People with MZIN are a high-risk group for anogenital cancers and subsequently may require tailored monitoring in specialist multi-disciplinary clinics. Centralisation of care and education for primary care providers may improve management. The development of guidelines which incorporate all clinical stakeholders are now needed to provide an international framework regarding the screening, diagnosis, treatment and future prevention of MZIN.

## Introduction

People with persistent high-risk (oncogenic) human papillomavirus (HPV) infection are at increased risk of high-grade squamous intraepithelial lesions (HSIL) and squamous cell carcinoma (SCC) of the anogenital region. The lower anogenital squamous terminology (LAST) consensus recommends that squamous intraepithelial lesions are defined as low-grade (LSIL) and high-grade (HSIL) of all anogenital sites [1]. This terminology is widely implemented by American societies. In many European countries, *intraepithelial neoplasia* (-IN, with an accompanying grade) is the most commonly used term for describing pre-malignant lesions of the anogenital region. *Multizonal* describes disease in multiple (two or more) zones, where a zone represents a distinct anatomical anogenital site.

Treatment of cervical, vulval and vaginal high-grade intraepithelial neoplasia (equivalent to HSIL) to prevent cancer is well established in gynaecological practice. Recently, the treatment of anal HSIL has been shown to prevent anal SCC [2]. It is, however, poorly understood that women*^1^ with vulval, vaginal or cervical intraepithelial neoplasia (VIN, VaIN, CIN) can also have perianal and/or anal intraepithelial neoplasia (AIN) and that this should be both considered and actively looked for. Whilst organised cervical screening has been a success in reducing cervical cancer, other HPV-related anogenital cancers continue to increase in incidence [3]. Although management of high-risk cervical lesions is well-established as a cancer prevention strategy, management of HSIL occurring at adjacent anatomical sites is less standardised.

Vulval cancer is a rare disease, with a world age-standardised incidence rate of 0.9/100,000 [4]. The incidence is higher in high income countries. In the UK, it affects 3/100,000 women per year [5], and HPV-associated vulval cancer is increasing, especially in younger women [6, 7]. Vulval intraepithelial neoplasia is diagnosed in 3.8/100,000 women/year [8] and classified as either differentiated VIN (dVIN) or vulvar high-grade squamous intraepithelial lesions (vHSIL). While vHSIL is HPV-dependent and accounts for almost 90% of cases, dVIN usually develops on the background of chronic dermatoses such as lichen sclerosus [9]. Vaginal cancer is extremely rare; in the UK there are around 250 cases diagnosed annually [10]. Anal squamous cell cancer has a world age-standardised incidence rate of 0.6/100,000 for women [4]. Some of the highest rates of anal cancer have been reported in Europe, including the UK [11], particularly in women, with an estimated age-standardised incidence rate of 3.1/100,000 in women versus 1.8/100,000 in men [12]. For women over the age of 50 in the UK, the incidence rate is 4–9/100,000 [12]. In the USA, the incidence of anal SCC overtook that of cervical SCC for the first time in 2014 and 2015 in Caucasian women over 75 and 65 years old, respectively [13]. Similarly, in England, in 2019 the incidence rate of both vulval and anal SCC exceeded the incidence rates of cervical SCC in Caucasian women over 55 years of age [14].

And also immunosuppresion is an important risk factor for all HPV-related neoplasia. In women living with HIV, the incidence of anal cancer is 22/100,000 [15]. It is difficult to obtain precise estimates of AIN incidence, given that the majority of preinvasive lesions are not recorded in national registries, however, trends suggest that the incidence of AIN is increasing, and like VIN, this trend is particularly pronounced in women younger than 60 years [16]. Meanwhile, although cervical cancer rates have decreased amongst vaccinated cohorts, and herd immunity effects have been observed [17], the latency period between HPV infection and peak incidence of anal and vulval SCC means that the full impact of vaccination will not be fully realised for 20–30 years [18].

MZIN is objectively rare, however patients with this condition present a significant management challenge to gynaecological services and the presence of anal HPV-related disease is often underestimated. For this reason, this document has been prepared by the British Society for Colposcopy and Cervical Pathology (BSCCP), in collaboration with members of the International Anal Neoplasia Society (IANS), the European Federation for Colposcopy (EFC) and British Society for the Study of Vulval and Vaginal Disease (BSSVD), with the aim of summarising existing national screening practices for multizonal anogenital cancers in women, defining the terminology for use in referring to MZIN, as well as best practice for diagnosis and assessment.

## Terminology: defining the umbrella term

The term multizonal to describe intraepithelial neoplasia affecting multiple anogenital sites is preferred to terms such as multifocal or multicentric disease or field effect, which in the past have been used interchangeably. Intraepithelial neoplasia may arise in the skin or mucosa on a background of non-HPV-related dermatoses (dVIN) or HPV-related disease. Slaughter et al. [19] first described the field effect of HPV, whereby daughter cells carried genetic alterations associated with HPV infection, however, the evidence for such monoclonal spreading is inconclusive. Now it is thought more likely that multiple different pathways contribute to the evolution of multizonal disease, including the persistence of high-risk HPV in multiple areas on the background of predisposing factors such as genetic risk and/or immunosuppression [20, 21]. Studies of HPV genetic variation have shown that it is possible that either a single infection, or alternatively several different HPV types cause can cause disease across multiple zones [19, 22].

**Fig. 1:**
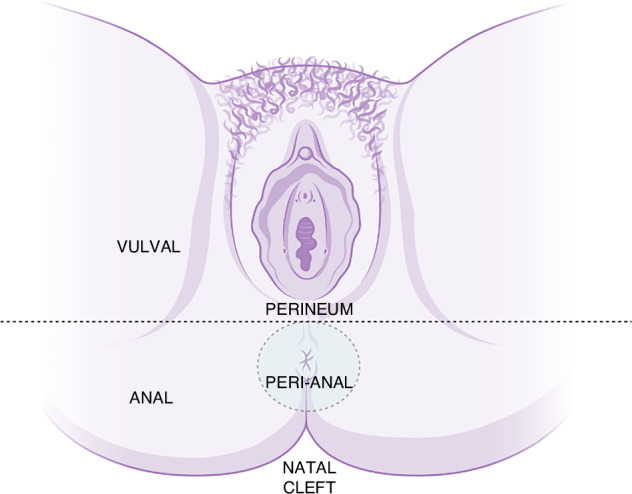
Diagram depicting specified zones. The anterior perianal area is defined as beginning halfway between the posterior border of the fourchette and the anterior anal margin (reference ANCHOR study).

The nomenclature provided in this article relates to MZIN, which is considered to involve more than one of the five zones in females: vulva, vagina, cervical, perianal and anal canal zones (Fig. 1). The perineum can be considered as part of the vulva however a separate definition of this anatomy allows for a more accurate description in the case of MZIN. We therefore define the perineum as the area beginning at the posterior fourchette and extending to the perianus. The same mechanisms may lead to an analogous scenario in men of penile, scrotal, perineal, perianal and anal canal. The natal cleft can be considered an extension of the perianal zone, but disease is often missed at this site. While MZIN may include either low-grade or high-grade IN, it is the early detection and treatment of high-grade IN of the anogenital zones which has the best evidence for prevention of cancer [23].

In terms of time frame, we define MZIN as either ‘synchronous’ or ‘metachronous’ disease. Synchronous refers to disease occurring in two different zones at the same time (with diagnoses made within 1 year). Disease diagnoses in differing anogenital zones, occurring outside of 12 months, is considered metachronous. The differential risk of cancer in synchronous versus metachronous disease requires further study. These women should still be considered at potentially greater risk of cancer and therefore referred for a specialist MZIN assessment.

## Screening of the anogenital tract

Screening for HPV-related disease outside of the cervix, for example, of vulva, vagina, anal, perianal and penoscrotal areas, is not currently recommended in the general population, as the *Wilson and Jungner* principles of effective screening [24] are not met. The incidence of these cancers is lower, and the efficacy of early detection and treatment of preinvasive lesions for reducing both the incidence and mortality from these cancers is less established. Screening recommendations should balance the anticipated benefits for early disease detection and treatment against potential harm, and resource implications need to be considered.

In most countries, screening recommendations for pre-invasive anogenital disease outside of the cervix has been limited to persons already diagnosed with neoplasia of a single anogenital site, or where resources allow, in pre-defined high-risk groups. Under English guidance [25], newly diagnosed HIV-positive persons are eligible for an initial colposcopy (visualisation of the cervix using magnification and dilute acetic acid), plus international guidelines have suggested digital anorectal assessment (DARE) with an anal HPV test and anal cytology, followed by annual screening, where resources permit [26].

The increased risk of anal cancer in immunosuppressed persons, including HIV, has been well-documented. Clifford et al. [15] identified several high-risk groups for anal cancer in a meta-analysis, including: people living with HIV; men who have sex with men; solid organ transplant recipients (SOTRs); patients with autoimmune disease; and women diagnosed with cervical cancer and HPV-related vulvovaginal cancers and precancers, including high-grade VIN, VaIN or CIN [15]. In the 2022 landmark ANCHOR study [2], a phase-3 trial where 4459 participants living with HIV who were diagnosed with biopsy-proven anal HSIL were randomised to treatment or active monitoring with high-resolution anoscopy (HRA), treatment was confirmed to be effective for anal cancer prevention. Subsequently, international guidelines have been published to recommend screening for AIN and anal cancer in high-risk groups where incidence exceeds ten times that of the general population, i.e. 17/100,000, which notably includes women with previous or current VIN, vulval SCC and SOTR [27]. In persons with a history of cervical/vaginal HSIL or cancer, perianal warts, persistent cervical HPV16, the incidence of anal cancers is reported as less than 10/100,000 [28], consequently the IANS recommendation for anal screening is this group (risk category B) was that they could be included for screening based on shared decision making, provided there is adequate capacity ofr HRA (*visualisation of the anal canal with magnification and dilute acetic acid*) [27].

Despite limited research, there is increasing recognition by specialists managing anogenital cancers that women with MZIN are a high-risk group for cancer and may require tailored monitoring and assessment of all anogenital zones [29]. Although there are currently no formal screening programmes, there are a few units within the UK and elsewhere that are performing multizonal assessments of the anogenital tract in one setting. However, most women with MZIN are still seen by multiple separate specialists. Improved definition of MZIN, as well as symptom recognition in primary care may improve early detection and appropriate referral. In addition, educating women about increased risk is essential for improving early detection.

Although several consensus guidelines exist for the management of HPV-related disease in different anatomical areas, guidance on the specific assessment and management MZIN is lacking. A complete colposcopic examination for an abnormal cervical screening test should include at least an assessment of the vulva, perineum and vagina [30]. In people at high risk of MZIN (and cancer), such as those living with HIV, SOTR or people with a history of cervical, vulval or vaginal cancer (including hysterectomised patients), complete assessment needs to be expanded to all zones, including the anus and perianus [2, 31]. There is also a need to recognise the significantly increased risks of cancer [29] in people with MZIN, and for assessment by clinicians with the appropriate skills. Nonetheless, more frequent assessments must be balanced within the context of the resources available, and the additional psychological and physical effects of more regular assessment, and potential for overtreatment. Centralisation of care may improve management. Education of patients with MZIN and their primary care providers regarding symptoms and risk is also important so that red flag symptoms presenting to primary care are acted upon in a timely manner. Furthermore, there is a need to establish one terminology across specialties, as well as the overlapping disease pathology across anatomical zones, which simultaneously recognises the differences between zones. These improvements would also facilitate further research into this complex and challenging condition.

## High-risk groups

**Fig. 2:**
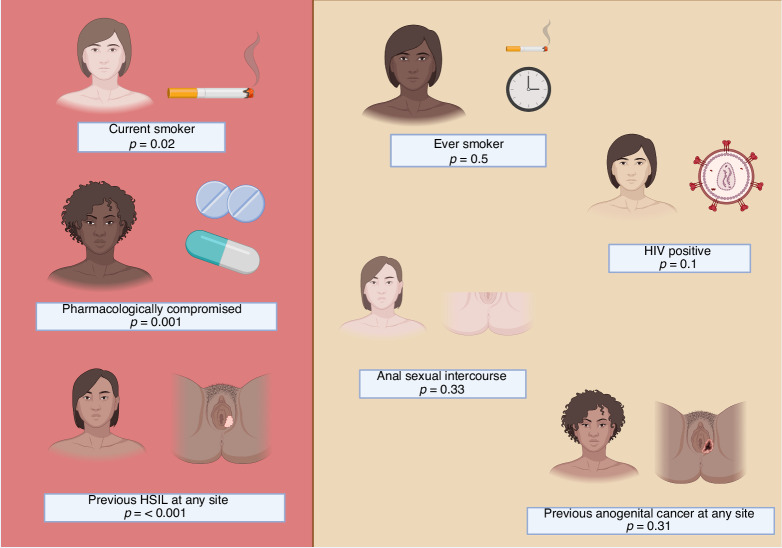
Risk factors associated with multizonal anogenital neoplasia (MZIN) diagnosis at first assessment and/or follow up. Based on a cohort with MZIN (*n* = 56) and without (*n* = 197). Taken from Albuquerque et al., BMC Cancer, 2021.

The definition of high-risk groups helps to identify those who may benefit from regular review in clinic-based screening. Based on a small study comparing synchronous MZIN (*n* = 56) and non-MZIN (*n* = 197) groups [29], MZIN diagnosis appears to be more common in women who are current smokers, those taking immunosuppressants and those with previous HSIL at any site. There were non-statistically significant differences between the two groups associated with ever smoking, anal sexual intercourse, HIV positivity, and previous anogenital cancer at any site (Fig. 2). In this study, 98% of women with MZIN had a history of either HSIL or cancer in one of the anogenital zones. The higher frequency of HSIL likely led to the stronger statistical association observed for HSIL, over the association seen for cancer.

## Assessment in the presence of symptoms of MZIN

While terminology for colposcopy is well-defined, terminology for MZIN remains unclear. The International Federation for Cervical Pathology and Colposcopy (IFCPC*^2^) terminology and colposcopy guidelines published in 2011 [32] have allowed enhanced reliability and reproducibility of clinical assessment findings during assessment of the cervix and vagina. Here, we expand the terminology specifically for MZIN. Most cases of intraepithelial neoplasia are asymptomatic which makes diagnosis challenging. Symptoms that patients can present with include:

- Persistent vulval or perianal itch and irritation.
- Persistent pain or tenderness in affected zones.
- Skin changes in the affected zones (discolouration, lump, erythema, excoriation, wet/weeping lesions, warts).
- Per rectal bleeding and anal pain or mucus discharge (although in this context a diagnosis of colorectal/anal malignancy must first be excluded).
- Anal fissures with atypical features (including atypical site i.e. not directly anterior or posterior); raised chronic fissures with palpable edges; new fissures in older patients (age 50+); any fissure not responding to six weeks of topical nitric oxide donor treatment (e.g. GTN 0.45%, Diltiazem 2%).
- Sensation of rectal mass or fullness.
- Postcoital or intermenstrual bleeding.

When women with symptoms of potential MZIN, (or from a high-risk group with any symptoms of anogenital IN) present in the primary care setting, or any secondary care setting without access to colposcopy and/or HRA, a thorough clinical assessment of all zones should be performed (Table 1).

**Table 1:** Terminology on the clinical assessment findings outside of Colposcopy/HRA setting.

Zone	Pattern
Inspection of vulva	The vulva encompasses the external female genital organs: mons pubis, outer (labia majora) and inner (labia minora) folds or lips, clitoris with clitoral hood, opening to the urethra and vagina (vestibule). The vulva adjoins the perineum. Inspection should include all areas.
Speculum examination of cervix and vagina	Inspect the cervix and vagina for any visible abnormality using the speculum and by slowly retracting the speculum.
Perianal inspection and DARE (Digital anorectal examination)	The perianus is defined as 5 cm from the anal margin except anteriorly when it is defined as halfway between the posterior border of the fourchette and the anterior anal margin [2] DARE should be performed according to IANS standards [26] to assess for a palpable anorectal lesion.

The diagnosis of MZIN in primary or secondary care can be challenging. Therefore, symptoms of MZIN or identification of potential MZIN warrant a full examination in a specialist setting (Table 2).

**Table 2:** Terminology for the clinical assessment findings of a MZA (colposcopy, vulvoscopy, vaginoscopy, HRA) in the specialist setting.

**Equipment required**	
• 3–5% acetic acid • Lugol’s iodine • Colposcope • Speculum • Proctoscope • Lithotomy leg supports	
**General considerations for assessment of MZIN**	
– Obtain adequate consent (verbal) with explanations of all areas. Consent may also include the use of medical photography to document the disease pattern for future monitoring and communication with specimens sent for histopathology. – Either begin with the cervix and then vagina, vulva, perianus and anal canal OR begin with site of active disease then proceed to the rest of the exam (if anal first, change gloves/clean acetic acid) in case of withdrawal of consent prior completion of MZA. – Assess the overall skin quality, anatomy and symptomatic areas highlighted by the woman herself. – Place the patient in lithotomy for assessment of the vulva, perianal skin, vagina and cervix with a colposcope providing magnification and good light. Assessment of the anal canal by DARE or HRA is performed in lithotomy or in the left lateral position. If in lithotomy, a table with the ability to tilt backwards by 30° is required (the Trendelenberg position). – Assessment of the vulval and perianal skin does not always involve the application of dilute acetic acid, however vaginoscopy, colposcopy and high resolution anoscopy requires the application of a 3 or 5% acetic acid solution often followed by the application of Lugol’s iodine. – Punch biopsies taken from the cervix and innermost anal canal do not necessarily require local anaesthetic. LA is used prior to taking biopsies from the other zones.	
**Detailed assessment by Zone**	
**Zone**	**Pattern**
Vulva	Vulvoscopy
	Examine the vulva from outer to inner, towards the mons pubis and groin.
	General assessment
	- Adequate or inadequate view of labia majora, labia minora, clitoral hood, vestibule, fourchette, perineum and perianal skin (see adjoined sections).
	Normal findings - Squamous epithelium (mature, atrophic), keratinised/non-keratinised epithelium. - Hyperkeratosis/Pallor/Erythema (generalised/localised). Abnormal findings - General principles: examine for erythema, hypo/hyperpigmentation, lichenification, plaques/patches/nodules/blisters/erosions and ulcers, diffuse genital oedema. - Grade 1 (minor). - Grade 2 (major). - Suspicious for invasion. - Non-specific—blisters, erosions, ulcers, diffuse genital oedema.
	Lesion identified: describe type/solitary/multiple/laterality and location, size (scale if over 7 mm).
	Miscellaneous findings:
	Non-HPV related VIN/Differentiated VIN; abscesses; non-HPV dermatological findings, excoriation, erythema;
	Groin lymph nodes should be assessed in the presence of a vulval or anal lesion
Perineum	General assessment
	Examine the area between vaginal introitus and anus
	Normal and abnormal findings
	Follow the assessment of the vulva for normal and abnormal findings given the keratinised squamous epithelium of the perineal skin
Vagina^a^	General assessment
	- Adequate or inadequate. - Includes fornices, lateral, anterior and posterior, upper and lower walls on withdrawal of speculum; introitus, (post-hysterectomy examine the upper vaginal sutured corners of the vault). - Squamous epithelium (Mature, atrophic).
	Abnormal findings
	– General principles. – Grade 1 (minor). – Grade 2 (major) – Suspicious for invasion – Non-specific
	Miscellaneous findings
	- Erosion, condyloma, polyp, cyst, endometriosis, inflammation, vaginal stenosis, congenital TZ.
Cervix	Colposcopy should be performed in the lithotomy position with the aid of a colposcope and 3% or 5% acetic acid, often followed by Lugol’s iodine. A cervical high-risk HPV test and cytology test should be taken in line with local screening policy or colposcopy follow-up.
	- Adequate or inadequate - SCJ visibility - TZ type 1, 2 or 3
	Normal colposcopic findings
	- Original squamous epithelium - Columnar epithelium; ectropy/ectropion - Metaplastic squamous epithelium; Nabothian cysts - Deciduous in pregnancy
	Abnormal colposcopic findings
	- General principles - Locations of the lesion - Size of the lesion: number of quadrants - Size of the lesion as percentage of the cervix - Grade 1 (minor) - Grade 2 (major) - Nonspecific: leukoplakia, erosions - Lugol’s staining: stained or nonstained
	Suspicious for invasion
	- Atypical vessels - Additional signs: fragile vessels, irregular surface, exophytic lesions, necrosis, ulceration, tumour or gross neoplasm
	Miscellaneous findings
	Congenital TZ, condyloma, polyp, inflammation, stenosis, congenital anomaly, post-treatment consequence, endometriosis.
Perianal	Examine the whole perianal area including the natal cleft using magnification (i.e. colposcope) usually with acetic acid
	General Assessment
	- Adequate or inadequate - DARE to assess for palpable lesions.
	Normal findings
	- Normal skin changes related to trauma, skin tags and visible haemorrhoids.
	Abnormal findings
	- Lesion identified: classify by colour, size, location, contour, surface patterns, vascularity and margins
	Suspicious for invasion
	- Atypical vessel, friability, irregular surface, exophytic, necrosis, ulceration, hard, palpable, obvious mass.
	Miscellaneous findings
	Condyloma, anal fissure and post treatment changes, infective ulcerations and lesions including Herpes Simplex Virus
Anal	HRA (Magnification with colposcope, acetic acid 5% plus or minus Lugol’s iodine) [27]: Should be carried out with proctoscope/anoscope, magnification via colposcope and application of 5% acetic acid and usually of Lugol’s iodine. Include proximal anatomical marking of the squamocolumnar junction; transitional zone if present; mid anal canal, dentate line and gland openings and distal anal canal.
	Can be performed in left lateral or lithotomy (with ‘head down’)
	If resources available, a swab for anal cytology (and/or anal high-risk HPV) should be taken first, prior to DARE [26]
	General Assessment
	- Adequate or inadequate—was entire squamocolumnar junction identified?
	Normal findings
	- Original squamous and columnar epithelium seen. - Metaplastic squamous epithelium; gland openings
	Abnormal findings
	- Lesion identified: classify by colour, size (number of octants), location, contour, surface patterns, vascularity, margins, Lugol’s staining: likely LSIL or HSIL, punctation, striations, mosaic pattern.
	Suspicious for invasion
	Atypical vessel, friability, irregular surface, necrosis, ulceration, palpable firm or hard lesions; obvious mass.
	Miscellaneous findings
	- Condyloma, anal polyp, post treatment changes, fissure; internal haemorrhoids.
Biopsies of suspicious areas	
Cervical punch biopsies do not usually require local anaesthetic. Internal anal canal biopsies at the transitional zone likewise, although local with adrenaline is often preferred to reduce bleeding. Biopsies of other areas should be taken with local anaesthesia in most cases: lidocaine 1% or 2% with adrenaline can be used; consider spray with topical 10% xylocaine prior; consider buffering with 8.4mEq sodium bicarbonate ratio 4:1 for sensitive areas eg vulva and distal anal canal. Consider Monsel’s solution (ferrous subsulphate) and/or silver nitrate for haemostasis where required.	

*MZA* multizonal assessment, *HRA* high-resolution anoscopy, *MZIN* multizonal intraepithelial neoplasia, *DARE* digital ano-rectal exam, *LA* local anaesthetic, *HPV* human papillomavirus, *VIN* vulval intraepithelial neoplasia, *SCJ* squamo-columnar junction, *TZ* transformation zone, *LSIL* low-grade squamous intraepithelial lesion, *HSIL* high-grade squamous intraepithelial lesion, *IFCPC* International Federation for Cervical Pathology and Colposcopy.

Cervix and Vagina adapted from Bornstein et al. 2012 [25].

^a^The presence of a neovagina, previous radiation, previous vulval reconstruction or other special cases may require the input of a subspecialist.

The patterns of HSIL in the anus and perianus are similar to those in the lower genital tract. However, at present there are few gynaecologists trained to conduct HRA to the standards presented in the IANS 2016 guidelines [26, 33]. Existing practitioners with an interest should be supported to formally train in HRA to carry out a full multizonal assessment (MZA) (see Table 2), with support from MZIN specialists where required.

In patients with evidence of MZIN, where local skills are lacking in HRA, referral to colorectal surgery for assessment can be considered if there are (a) any symptoms of anal or perianal disease as outlined above, (b) any abnormality on the DARE or (c) the need for screening given the burden of disease elsewhere. The anal cancer screening guidance from IANS [27] suggests that annual DARE for high-risk groups is carried out by trained practitioners in regions where HRA is lacking [34]. Nonetheless, training in HRA is encouraged due to the recognition of the need for this service, particularly for women with MZIN.

Oropharyngeal HPV-related disease is rare and particularly so in women [35], with no currently available screening programme. Clinicians managing women with MZIN should remain aware of its common presentations. These include persistent throat soreness, a lump in the throat or neck and difficulty swallowing [36]. Referral to the relevant clinical specialty should be prompt.

## Other considerations

To our knowledge, there are no studies exploring the psychological experience or needs of women with MZIN. There are negative psychological consequences associated with HPV-related disease, which may be heightened in MZIN, including stigma, fear of disease progression, reduced quality of life, sexual dysfunction and dissatisfaction with partner relationships [37–39]. MZIN patients may have extensive disease, and treatment can have particularly disfiguring results. It is important that the informational needs of women with MZIN are met and that patient concerns about MZIN and any associated treatment are acknowledged and supported [40].

Clinicians should consider the social determinants of health which have consequential effects on HPV-associated cancer incidence. Anal cancer has a higher incidence in lower socioeconomic groups [41]. Women from ethnic minority groups may have a lower awareness of their cancer risk, and lower uptake of screening or HPV vaccination [42]. HPV vaccination uptake can substantially reduce the incidence of CIN3 and cervical cancer across differing socioeconomic groups, including those demonstrating domains of deprivation [43]. Globally, there remains significant health inequalities for both cervical screening [44] and HPV vaccination coverage with disparity persisting for lower-income countries [42, 45]. Tackling misinformation is needed, particularly for parents who need to be informed about the protective effects of timely HPV vaccination for their adolescent children [46]. Black African or Black Caribbean women have been shown to be more likely to be diagnosed with anal SCC at a younger age and advanced stage [41]. Cultural barriers and the risk of stigma may result in hesitancy when discussing symptoms pertaining to the anogenital tract. There remains a gender bias that results in discrimination based on age, race, ethnicity, socio-economic status, sexual orientation, and gender identity that can heighten barriers in accessing healthcare services [47]. We encourage the use of inclusive language and access to translatable information for non-native English-speaking individuals.

## Summary

This paper forms the first in a series of consensus papers referring to MZIN with the aim of providing consensus guidelines describing assessment and management. Through cross-specialty collaboration, we highlight the preference of the terminology of MZIN over other terms, and the definition of MZIN as either synchronous disease (occurring in more than two zones within 12 months) or metachronous (outside of 12 months).

While there are an increasing number of guidelines developed for anogenital neoplasia, MZIN remains poorly defined resulting in a wide variation in the assessment and management of such patients. As MZIN is rare, and its complexity requires multiple specialised skillsets, management by multidisciplinary teams is more appropriate and may optimise patient experience. Treatment should be individualised and tailored to the location and extent of disease, whilst adapting to the characteristics and risk factors of the woman, any special considerations, as well as previous history of HSIL or cancer.

This group intends to develop a set of consensus guidelines on the recommendations for the management of MZIN, including defining high-risk groups for clinic-based screening, defining the optimal tools for diagnosis and assessment of disease burden, issues related to training and quality control, identification of MZIN in primary care and psychosocial support.

## Future aims

- Development of consensus guidelines is needed, with multidisciplinary agreement on how best to deliver services for women at risk of or diagnosed with MZIN, especially in the context of previous anogenital HSIL.
- Centralisation of care should be considered for patients with MZIN, this warrants further attention by relevant international societies
- Enroling patients with MZIN into national research studies could allow for identification of specific clinical features or distinct biomarkers which predict MZIN versus single-site disease
- It is recognised that MZIN carries a significant psychological burden, further investigation into psychological sequelae of the disease is needed to determine how these women can be best supported and treated.

## Footnotes

1 1. Within this document we use the term women. However, we recognise that it is not only people who identify as women who access women’s health services to maintain their gynaecological health, which may also include non-binary persons and transmen who were registered female sex at birth.

2 The IFCPC since 2024 has undergone a name change to the International Federation for Cancer Prevention and Colposcopy.
